# Psoriatic Arthritis and Diabetes: A Population-Based Cross-Sectional Study

**DOI:** 10.1155/2013/580404

**Published:** 2013-06-17

**Authors:** Jacob Dreiher, Tamar Freud, Arnon D. Cohen

**Affiliations:** ^1^Clalit Health Services, Faculty of Health Sciences, Ben-Gurion University of the Negev, 84150 Beer-Sheva, Israel; ^2^Siaal Research Center for Family Medicine and Primary Care, Faculty of Health Sciences, Ben-Gurion University of the Negev, Beer-Sheva, 84150, Israel

## Abstract

*Background*. Diabetes has been associated with psoriasis, but little is known about the association between psoriatic arthritis and diabetes. *Methods*. Patients diagnosed with psoriatic arthritis by a rheumatologist were compared to age- and sex-matched patients without psoriatic arthritis regarding the prevalence of diabetes in a population-based cross-sectional study using logistic multivariate models. The study was performed utilizing the medical database of Clalit, the largest healthcare provider organization in Israel. *Results*. The study included 549 patients with psoriatic arthritis ≥21 years and 1,098 patients without psoriatic arthritis. The prevalence of diabetes in patients with psoriatic arthritis was increased as compared to the prevalence in patients without psoriatic arthritis (15.3% versus 10.7%, *P* value = 0.008). The difference was prominent among females (18.7% versus 10.3%, *P* < 0.001) but not among males (11.2% in patients with and without psoriatic arthritis, *P* = 1.000). In a multivariate analysis, psoriatic arthritis was associated with diabetes among females (OR = 1.60, 95% CI: 1.02–2.52, *P* = 0.040) but not among males (OR = 0.71, 95% CI: 0.42–1.22, *P* = 0.213). *Conclusion*. Our study suggests a possible association between psoriatic arthritis and diabetes in women. Women with psoriatic arthritis might be candidates for diabetes screening.

## 1. Introduction

 Psoriatic arthritis (PsA) is a chronic inflammatory arthropathy characterized by the combined presence of psoriasis and arthritis. It affects 5–8% of psoriasis patients and 0.05–0.24% of the general Caucasian population [1, 2]. Many patients develop progressive joint damage and disability, with a significant impact on the patient's functional status and quality of life, and a reduced life expectancy related to cardiovascular and respiratory causes [3, 4].

Psoriasis was reported to be associated with cardiovascular risk factors [1, 5–18]. PsA also appears to be associated with an increased cardiovascular mortality and morbidity. Several factors including smoking, dyslipidemia, hypertension, increased fibrinogen level, hypercoagulability, and decreased physical activity might explain the enhanced cardiovascular risk in patients with PsA. Under-treatment of cardiovascular morbidity also may contribute to the higher cardiovascular risk [19]. Markers of disease activity seem to be associated with increased cardiovascular mortality [4]. Nevertheless, the evidence base to support the hypothesis of increased cardiovascular risk in PsA is much less definitive than in other inflammatory joint diseases such as rheumatoid arthritis [20].

Previous studies have reported an increased prevalence of diabetes in patients with psoriasis [1, 5–17, 21]. Diabetes was found in other systemic inflammatory disorders such as systemic lupus erythematosus [22] and rheumatoid arthritis [12, 23]. Patients with PsA were found to have higher fasting glucose than patients with RA (30% versus 16% [24]). A similar association between PsA and diabetes was also noted [3, 15, 18, 19, 21, 25].

We have previously described the association between psoriasis and diabetes [11]. The purpose of the current study was to assess the association between PsA and diabetes utilizing the large medical dataset of Clalit, the largest healthcare provider organization in Israel. 

## 2. Subjects and Methods

For the current study, data mining techniques utilizing the database of Clalit were used. Clalit serves a population of approximately 4,100,000 enrollees. A comprehensive computerized database with continuous real-time input from pharmaceutical, medical, and administrative computerized operating systems facilitates epidemiological studies such as the current analysis.

In recent years, we have used Clalit's database to study disease associations in patients with psoriasis; the methodology has been previously described [11]. In the current study, patients were defined as having PsA when a diagnosis of PsA was documented at least twice in the medical records registered by a Clalit's rheumatologist in the community. The comparison group (patients without PsA) was randomly selected from the list of Clalit members, excluding patients for whom a diagnosis of PsA was made at least once and was frequency-matched to patients with PsA regarding sex and age, age being matched within 8 age groups (18–24, 25–34, 35–44, 45–54, 55–64, 65–74, 75–84, and 85 or older). Data available from the Clalit database included age, sex, smoking status, and socioeconomic status. The diagnoses of chronic diseases were extracted from Clalit's chronic diseases registry, which is based on data withdrawn from hospital and primary care physicians' reports. The registry is validated by primary physician confirmation of registered diagnoses of chronic diseases. The validity of diagnoses in the register was previously estimated and found to be high for important chronic diagnoses, including diabetes [26, 27].

The study was approved by the institutional review board of Soroka University Medical Center. Publication of the data was approved by the institutional committee of Clalit's general management.

The distribution of background factors was compared between patients with and without PsA using the chi-square test for categorical variables and *t*-test for age. The proportions of patients with diabetes were compared between the study groups in the entire study sample as well as in a stratified analysis based on age, sex, and other subgroups using the chi-square test. The presence of effect modification was identified using the Breslow-Day test. Odds ratios and 95% confidence intervals are presented as well. A logistic regression model was used to measure the association between PsA and diabetes in a multivariate analysis. To test the accuracy of the model, the area under the ROC curve (receiver operating characteristics) as well as the Hosmer-Lemeshow test for the goodness-of-fit were calculated. Statistical analysis was performed using SPSS software, version 21.

## 3. Results

The study included 549 patients with PsA over the age of 21 years and 1,098 age and sex frequency-matched patients without PsA. Descriptive analyses of the characteristics of patients with and without PsA appear in Table 1. Patients with PsA were of lower socioeconomic status, more likely to be current smokers, and more likely to be diagnosed with hypertension, dyslipidemia, and obesity (Table 1). The prevalence of diabetes in patients with PsA was increased as compared to the prevalence in patients without PsA (15.3% versus 10.7%, OR = 1.50, 95% CI 1.11–2.03, *P* value = 0.008). 

A statistically significant effect modification by sex was noted (*P* = 0.029 in the Breslow-Day test). Therefore, we analyzed the data separately for females and males. Descriptive statistics for males and females appears in Table 2. The association between diabetes and PsA was statistically significant among females (18.7% versus 10.3%, OR = 1.99, 95% CI: 1.35–2.95, *P* < 0.001, Table 2). In a stratified analysis, the association was a significant among patients <65 years, among nonsmokers, and among nonobese females, and patients without hypertension and without dyslipidemia (Table 3). In contrast, the association between diabetes and PsA among males was not statistically significant (11.2% in both patients with and without PsA, OR = 1.00, 95% CI: 0.61–1.62, *P* = 1.000, Table 2). In a stratified analysis, the association was not significant in any subgroup in males (Table 4).

In a multivariate analysis, diabetes was associated with PsA among females after controlling for age, socioeconomic status, obesity, smoking, hypertension, and dyslipidemia (OR = 1.60, 95% CI: 1.02–2.52, *P* = 0.040, Table 5). The model for females was found to have a high goodness-of-fit (area under the ROC curve: 0.831, *P* value for the Hosmer Lemeshow test: 0.783). In contrast, diabetes was not associated with PsA among males after controlling for the same confounders (OR = 0.71, 95% CI: 0.41–1.22, *P* = 0.213, Table 5). The model for males was also found to have a high goodness-of-fit (area under the ROC curve: 0.840, *P* value for the Hosmer-Lemeshow test: 0.551).

## 4. Discussion

In the present study, female patients with PsA were more likely to be diagnosed with diabetes than age- and sex-matched patients without psoriasis. This association was statistically significant and was demonstrated even after controlling for potential confounders, including age and obesity. In contrast, the association between PsA and diabetes was not noted among males. 

Several autoimmune rheumatic diseases, including systemic lupus erythematosus, rheumatoid arthritis, and PsA were found to be associated with risk factors for atherosclerosis, including hypertension, hyperlipidemia (increased total cholesterol and low-density lipoprotein cholesterol) hyperhomocysteinemia, and diabetes [3, 12, 24, 28]. 

Previous studies have reported the association between psoriasis and diabetes [1, 5–17], as well as between PsA and diabetes [3, 4, 21, 25, 29]. In a small study from Israel, the prevalence of diabetes in PsA patients was higher than among controls, though the difference was not statistically significant (2/30 versus 1/30, *P* = 1.00) [4]. Han et al. [3] compared cardiovascular risk factors between patients with PsA, rheumatoid arthritis and ankylosing spondylitis, and age- and sex-matched controls. The prevalence of diabetes was higher among PsA patients than among controls (11.3% versus 7.3%, *P* < 0.01, prevalence ratio = 1.5, 95% CI: 1.4–1.7). No separate analysis for females and males was performed [3]. 

In a relatively larger study from Hong Kong [25], PsA patients were more likely than controls to have diabetes and had higher fasting glucose levels. These differences persisted after controlling for body-mass index (BMI) but lost statistical significance after controlling for C-reactive protein (CRP) levels, as a marker of inflammation. However, higher insulin levels and a greater degree of insulin resistance were noted among PsA patients, and these remained significant even after controlling for BMI and CRP [25].

In a recent study from Spain, patients with psoriasis had an increased prevalence of type 2 diabetes than controls (12.0% versus 6.1%, OR = 2.11, 95% CI: 1.59–5.20). Furthermore, among patients with psoriasis, the presence of PsA was associated with type 2 diabetes even after controlling for age, dyslipidemia, hypertension, BMI, family history, and age at onset (OR = 2.16, 95% CI: 1.19–3.90) [21]. A cohort study of patients with psoriasis and/or PsA reported a hazard ratio of 1.4 (95% CI: 1.3–1.5) for diabetes [29].

In the present study, the association between PsA and diabetes was noted only among women. How can this difference be explained? There are many sex differences in diabetes, including risk factors. Several factors could play a role. These include the mitochondrial homeostasis, the redox state, and some specific genes. Other studies claim that metabolic differences could be pivotal [30]. 

Some of the detected gender differences could be attributed to sexual hormones. For example, women are less insulin sensitive during the luteal phase of the menstrual cycle and in pregnancy. Estrogen deficiency, as occurs at menopause, may contribute to the elevated risk of diabetes for women. Oral contraceptives use may be related to reduced insulin sensitivity (about 40%) if compared with women with intact hormonal cycles [30]. Androgen status and insulin sensitivity seem also to be related. In hyperandrogenic conditions, such as polycystic ovarian syndrome, a significant increase in glucose intolerance, insulin resistance, metabolic syndrome, and type 2 diabetes has been found. Sexual hormones also affect the growth hormone/insulin-like growth factors axis, a key endocrine modulator of postnatal growth and metabolism [30]. 

 Women have a significantly higher fasting leptin, heart rate, and cardiac sympathetic activity and lower insulin sensitivity [30]. Women have higher levels of adiponectin (a hormone which is secreted by adipose tissue and regulates glucose metabolism) compared with men [31].

Levels of proinflammatory markers, such as hs-CRP and Interleukin(IL)-1 receptor antagonist, were previously reported to be significantly higher among women with metabolic syndrome compared with men with metabolic syndrome. In contrast, no gender difference in these markers between men and women was observed in subjects without metabolic syndrome [31]. These data suggest that chronic inflammation might have a stronger role in the pathogenesis of diabetes in women [30]. 

It has been suggested that some sex differences in diabetes could be due to gender differences in stress response. During stress reaction, the central nervous system stimulates the increase in circulating levels of catecholamines, glucocorticoids, and growth hormone resulting in energy-mobilizing effects that can be deleterious to the control of blood glucose [30]. A possible explanation might be that female patients with PsA in Israel are more likely to receive corticosteroids. Since the design of the study did not allow to differentiate type 1 or type 2 diabetes and did not include data on medical therapy for PsA, we could not rule out this mechanism.

 Several mechanisms could explain the association between PsA and diabetes. Psoriasis and PsA, the metabolic syndrome and atherosclerosis, are all characterized by an inflammatory process driven by Th1 cytokines [32]. Cytokines involved in psoriasis, PsA, and diabetes include tumor necrosis factor-alpha (TNF-*α*), IL-1, and IL-6. A chronic inflammatory state leads to the production of truncal fat adipokines (e.g., TNF-*α*, IL-6, and IL-8) which increase insulin resistance and endothelial dysfunction. These adipokines have been implicated in the developments of insulin resistance and type 2 diabetes. They drive the pathogenesis, maintenance, and intensity of psoriasis [16, 22]. Serum levels of resistin, another adipokine, were found to correlate with disease severity in psoriasis [33, 34].

TNF-*α* is a cytokine active in psoriasis and PsA that deserves special attention. TNF-*α* induces nuclear transcription factor-*κ*B (NF-*κ*B) activation and leads to oxidative stress, which exacerbates pathological processes leading to glucose intolerance and insulin resistance [35, 36]. Animal studies have shown that administration of TNF-*α* results in insulin resistance and that the lack of TNF-*α* protect against obesity-induced insulin resistance [16]. Some TNF-*α* blockers are reported to improve insulin resistance. In two small studies, psoriasis patients treated with etanercept demonstrated a decrease in fasting serum insulin levels. Infliximab was reported to improve diabetes control in a PsA patient, with worsening of diabetes control after infliximab was discontinued. On the other hand, adalimumab was reported to cause hyperglycemia in a patient with psoriasis, PsA, and type 2 diabetes. Chronic inflammation in psoriasis leads to increased insulin-like growth factor II, which promotes epidermal proliferation and is also linked to diabetes [16, 37]. It has been recently suggested that treatment with TNF-inhibitors or hydroxychloroquine may reduce the risk of diabetes in both psoriasis and PsA [18].

Genetic associations are also plausible. The psoriasis susceptibility loci *PSOR2*, *PSOR3*, and *PSOR4* are also associated with susceptibility loci for diabetes. Individual genes associated with psoriasis, such as CDKAL1, are also associated with diabetes [38–41]. In a genome-wide association study, psoriasis and PsA were associated with single-nucleotide polymorphism (SNPs) in Major Histocompatibility Complex genes, as well as in genes associated with IL-12, IL-12 receptor B2, and IL-23 receptor. A novel finding in this study was an association with several genes for IL-2 and IL-21 in chromosome 4q27, a region where SNPs associated with celiac disease and diabetes type 1 were also reported [42].

Another explanation to the association between PsA and diabetes may be related to unhealthy lifestyle, including an increased caloric intake, alcohol consumption, stress, and decreased exercise due to psoriasis symptoms, joint pain, or stigmatization in psoriasis and PsA patients, leading to diabetes [25]. Both psoriasis and diabetes are known to be associated with obesity and smoking [15]. Patients with psoriasis have an increased amount of visceral fat and are more likely to have nonalcoholic fatty liver disease (NAFLD) than BMI-matched controls. This is important because NAFLD is a recognized risk factor for diabetes [18]. In the present study, obesity and smoking were more prevalent among PsA patients than among patients without PsA. Obesity was associated with diabetes in both sexes, and smoking was associated with diabetes in males only. However, the association reported in the present study among females persisted after controlling for obesity.

The association between PsA and diabetes might have therapeutic implications. In a small series of 10 patients, treatment with pioglitazone, an insulin-sensitizing drug which is a thiazolidinedione drug used for the treatment of type 2 diabetes, led to significant improvement in several parameters, including tender and swollen joint counts, physician's global assessment, and patient's assessment of disability [43].

Studies which evaluated the association between PsA and insulin resistance have been criticized by Mallbris et al. [15] for having a small size and informal design, being prone to selection bias, and failure to control for smoking status [15]. In the present study, we tried to overcome these pitfalls by having a large number of cases, utilizing a formal cross-sectional design, controlling for smoking as well as obesity in the multivariate models. However, some study limitations persist.

The current study is limited by the lack of information on PsA severity and other risk factors (e.g., physical activity, age at onset, family history of psoriasis, or PsA). The directionality of the association cannot be established by the study design. Validation of PsA diagnosis was not available for each case; however, we included only cases where a diagnosis of PsA was recorded twice. Furthermore, we only included cases diagnosed by a rheumatologist, rather than a primary care physician. While this fact probably increased the specificity of the diagnosis, sensitivity might have been reduced and it is possible that PsA cases included in the study represent the more severe cases (referred to a rheumatologist). 

To conclude, the present study suggests a possible association between PsA and diabetes in women. Women with PsA were more likely to be diagnosed with diabetes than age- and sex-matched patients without PsA, while no association between PsA and diabetes was noted among men. Recently, screening using hemoglobin A1c for psoriasis patients with overweight or obesity has been suggested [18]. We suggest that women with PsA might therefore be candidates for diabetes screening.

## Figures and Tables

**Table 1:** Descriptive characteristics of the study population (*N* = 1,647).

Characteristic	Psoriatic arthritis patients (*N* = 549)	Patients without psoriatic arthritis (*N* = 1,098)	*P* value
Male	249 (45.4%)	498 (45.4%)	1.000
Age (years)			
Mean ± SD	53.8 ± 14.1	54.2 ± 14.7	0.599
Median	55	56	
Range	21–92	21–101	
SES (*N* = 1625)			
Low	193 (35.7%)	319 (29.4%)	**<0.001**
Intermediate	237 (43.9%)	436 (40.2%)	
High	110 (20.4%)	330 (30.4%)	
Hypertension	189 (34.4%)	281 (25.6%)	**<0.001**
Dyslipidemia	261 (47.5%)	436 (39.7%)	**0.002**
Current smoker	125 (22.8%)	191 (17.4%)	**0.006**
Obesity	129 (23.5%)	176 (16.0%)	**<0.001**
Diabetes	84 (15.3%)	118 (10.7%)	**0.005**

SES: socioeconomic status.

**Table 2:** Descriptive characteristics of the study population, by gender (*N* = 1,647).

	Females (*N* = 900)			Males (*N* = 747)		
Characteristic	Psoriatic arthritis patients (*N* = 300)	Patients without psoriatic arthritis (*N* = 600)	*P* value	Psoriatic arthritis patients (*N* = 249)	Patients without psoriatic arthritis (*N* = 498)	*P* value
Age (years)						
Mean ± SD	54.9 ± 14.4	54.4 ± 13.9		53.4 ± 14.9	53.0 ± 14.3	
Median	56	58	0.422	52	53	0.369
Range	22–92	21–97		21–92	23–101	
SES (*N* = 1625)						
Low	104 (33.5%)	160 (27.0%)		89 (36.0%)	159 (32.3%)	
Intermediate	130 (44.4%)	242 (40.8%)	**<0.001**	107 (43.3%)	194 (39.4%)	0.083
High	59 (20.1%)	191 (32.2%)		51 (20.6%)	139 (28.3%)	
Hypertension	110 (36.7%)	159 (26.5%)	**<0.001**	79 (31.7%)	122 (24.5%)	**0.036**
Dyslipidemia	147 (49.0%)	266 (44.3%)	**0.002**	114 (45.8%)	170 (34.1%)	**0.002**
Current smoker	55 (18.3%)	81 (13.5%)	**0.006**	70 (28.1%)	110 (22.1%)	0.070
Obesity	85 (28.3%)	109 (18.2%)	**<0.001**	44 (17.7%)	67 (13.5%)	0.137
Diabetes	56 (18.7%)	62 (10.3%)	**<0.001**	28 (11.2%)	56 (11.2%)	1.000

SES: socioeconomic status.

**Table 3:** Psoriatic arthritis and diabetes among females (*N* = 900).

Subgroups	*N*	Diabetes in psoriasis arthritis patients (*N* = 300)	Diabetes in patients without psoriatic arthritis (*N* = 600)	OR (95% CI)	*P* value
All	900	56 (18.7%)	62 (10.3%)	**1.99 (1.34–2.95)**	**<0.001**
Age					
20–44	222	3 (4.1%)	0 (0%)	**∞ (1.62–∞)**	**0.036**
45–64	453	32 (21.2%)	34 (11.3%)	**2.12 (1.24–3.60)**	**0.005**
65+	225	21 (28.0%)	28 (18.7%)	1.69 (0.88–3.25)	0.110
SES (*n* = 886)					
Low	264	21 (20.2%)	15 (9.4%)	**2.45 (1.19**–**5.01)**	**0.012**
Intermediate	372	23 (17.7%)	30 (12.4%)	1.51 (0.84–2.75)	0.164
High	250	11 (18.6%)	16 (8.4%)	**2.51 (1.09**–**5.76)**	**0.026**
Hypertension					
No	631	20 (10.5%)	22 (5.0%)	**2.24 (1.19**–**4.72)**	**0.010**
Yes	269	36 (32.7%)	40 (25.2%)	1.45 (0.84–2.48)	0.175
Dyslipidemia					
No	487	15 (9.8%)	4 (1.2%)	**8.97 (2.92**–**27.6)**	**<0.001**
Yes	413	41 (27.9%)	58 (21.8%)	1.39 (0.87–2.21)	0.165
Current smoker					
No	764	48 (19.6%)	53 (10.2%)	**2.14 (1.40**–**3.28)**	**<0.001**
Yes	136	8 (14.5%)	9 (11.1%)	1.36 (0.49–3.78)	0.552
Obesity					
No	706	27 (12.6%)	34 (6.9%)	**1.93 (1.13**–**3.29)**	**0.014**
Yes	194	29 (34.1%)	28 (25.7%)	1.50 (0.80–2.79)	0.201

SES: socioeconomic status.

**Table 4:** Psoriatic arthritis and diabetes among males (*N* = 747).

Subgroups	*N*	Diabetes in psoriasis arthritis patients (*N* = 249)	Diabetes in patients without psoriatic arthritis (*N* = 498)	OR (95% CI)	*P* value
All	747	28 (11.2%)	56 (11.2%)	1.00 (0.61–1.62)	1.000
Age					
20–44	204	3 (4.4%)	3 (2.2%)	2.05 (0.40–10.5)	0.403
45–64	387	11 (8.5%)	33 (12.8%)	0.64 (0.31–1.31)	0.213
65+	156	14 (26.9%)	20 (19.2%)	1.55 (0.70–3.39)	0.273
SES (*n* = 739)					
Low	248	11 (12.4%)	17 (10.7%)	1.18 (0.52–2.64)	0.691
Intermediate	301	13 (12.1%)	23 (11.9%)	1.03 (0.49–2.13)	0.940
High	190	4 (7.8%)	16 (11.5%)	0.65 (0.20–2.06)	0.465
Hypertension					
No	546	10 (5.9%)	24 (6.4%)	0.92 (0.42–1.97)	0.823
Yes	201	18 (22.8%)	32 (26.2%)	0.83 (0.42–1.61)	0.581
Dyslipidemia					
No	463	5 (3.7%)	8 (2.4%)	1.54 (0.49–4.79)	0.454
Yes	284	23 (20.2%)	48 (28.2%)	0.64 (0.36–1.14)	0.124
Current smoker					
No	567	13 (7.3%)	30 (7.7%)	0.94 (0.47–1.84)	0.844
Yes	180	15 (21.4%)	26 (23.6%)	0.88 (0.42–1.82)	0.731
Obesity					
No	636	20 (9.8%)	41 (9.5%)	1.03 (0.58–1.81)	0.922
Yes	111	8 (18.2%)	15 (22.4%)	0.77 (0.29–2.01)	0.593

SES: socioeconomic status.

**Table 5:** Psoriatic arthritis and diabetes—logistic regression models.

Variable	OR (95% CI)	*P* value
Logistic regression model for females (*N* = 886)		

Psoriatic arthritis	**1.60 (1.02–2.52)**	**0.040**
Age (per year)	1.02 (0.99–1.04)	0.182
Obesity	**3.12 (1.98**–**4.90)**	**<0.001**
Hypertension	**2.59 (1.58**–**4.22)**	**<0.001**
Dyslipidemia	**4.57 (2.54**–**8.24)**	**<0.001**
Smoking	**0.51 (0.26**–**0.98)**	**0.043**
Low SES (versus high)	1.71 (0.91–3.18)	0.091
Intermediate SES (versus high)	1.25 (0.71–2.17)	0.432

Logistic regression model for males (*N* = 739)		

Psoriatic arthritis	0.71 (0.41–1.22)	0.213
Age (per year)	**1.02 (0.99**–**1.04)**	**0.136**
Obesity	1.44 (0.78–2.65)	0.243
Hypertension	**1.88 (1.04**–**3.39)**	**0.035**
Dyslipidemia	**6.89 (3.54**–**13.4)**	**<0.001**
Smoking	**3.17 (1.95**–**5.15)**	**<0.001**
Low SES (versus high)	1.40 (0.70–2.78)	0.337
Intermediate SES (versus high)	1.17 (0.62–2.22)	0.621

SES: socioeconomic status.
